# Ancient and Emerging Nanostructures for Innovations to Fight Head and Neck Cancer

**DOI:** 10.3390/cells15040339

**Published:** 2026-02-13

**Authors:** Nina Kummer, Ömür Acet, Burcu Önal Acet, Mike Blueggel, Aya Khamis, Désirée Gül, Shirley K. Knauer, Roland H. Stauber

**Affiliations:** 1 Department of Otorhinolaryngology Head and Neck Surgery, Nanobiomedicine/Molecular and Cellular Oncology, University Medical Center Mainz, Langenbeckstraße 1, 55131 Mainz, Germanyguel@uni-mainz.de (D.G.); 2 Vocational School of Health Science, Pharmacy Services Program, Tarsus University, Tarsus 33400, Türkiye; 3 Chemistry Department Faculty of Arts and Science, Aksaray University, Aksaray 68100, Türkiye; 4 Institute for Molecular Biology II, Center of Medical Biotechnology (ZMB), University of Duisburg-Essen, Universitätsstrasse 5, 45141 Essen, Germany; mike.blueggel@uni-due.de (M.B.);; 5 Klinik für Mund-, Kiefer- und Gesichtschirurgie—Plastische Operationen, University Medical Center Mainz, Langenbeckstraße 1, 55131 Mainz, Germany

**Keywords:** HNSCC, ancient nanostructures, emerging nanostructures, nanobodies, engineered exosomes, DNA origami, stimuli-responsive nanoparticles

## Abstract

**Highlights:**

**What are the main findings?**

- Head and neck squamous cell carcinoma (HNSCC) is characterized by a highly resistant tumor microenvironment involving hypoxia, immune suppression, and stromal remodeling.
- Emerging nanostructures—including nanobodies, engineered exosomes, DNA origami, and (stimuli-)responsive nanoparticles—enable precise, targeted modulation of HNSCC pathobiology.

**What are the implications of the main findings?**

- Targeted modulation of the tumor and its microenvironment via advanced nanostructures offers a promising strategy to fight HNSCC.
- For clinical translation, critical challenges remain including ensuring safety, controlled responsiveness, and overall feasibility, which must be addressed to enable precision oncology approaches.

**Abstract:**

Head and neck squamous cell carcinoma (HNSCC) remains a major global health challenge due to its aggressive behavior, late-stage diagnosis, and high incidence of therapy resistance. At the cellular level, these clinical limitations are driven by profound alterations in oncogenic signaling, stress adaptation, DNA damage response pathways, and immune regulation within the tumor microenvironment. Advances in nanotechnology offer powerful opportunities to address these challenges by enabling targeted interference with cellular processes that govern tumor growth, survival, and therapy resistance. “Ancient” (i.e., established, long-studied) nanostructures, including mineral-based nanoparticles, natural biopolymers, and plant-derived nanovesicles, provide inherently biocompatible and bioactive platforms capable of modulating cellular signaling, redox balance, and immune responses. In parallel, emerging nanosystems—such as nanobodies, engineered exosomes, DNA origami, and stimuli-responsive smart nanoparticles—allow precise molecular targeting, controlled cargo release, and direct manipulation of intracellular pathways and intercellular communication. This manuscript synthesizes historical and contemporary developments in nanostructure design, highlighting how the integration of ancient materials with advanced nanotechnology can reshape therapeutic strategies for HNSCC. By targeting key cellular and microenvironmental processes, including DNA damage response signaling, redox homeostasis, immune regulation and stress-adaptive survival mechanisms, rather than drug delivery alone, these integrated nano-platforms offer promising avenues to overcome resistance mechanisms, reprogram the tumor microenvironment, and improve therapeutic precision and patient outcomes.

## 1. Introduction

### 1.1. Head and Neck Squamous Cell Carcinoma

**Figure 1:**
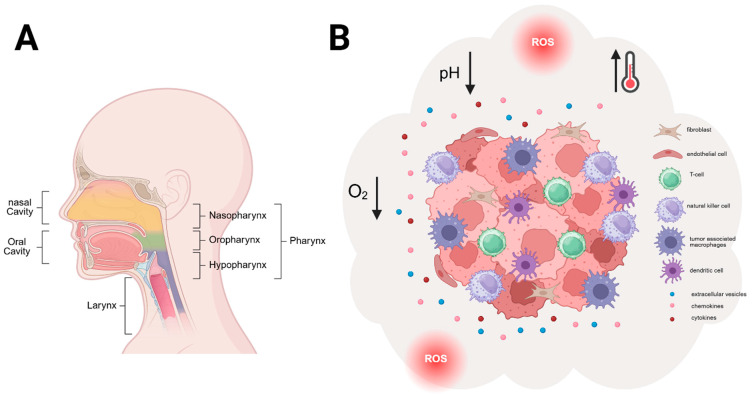
Localization and tumor microenvironment of HNSCC. (**A**) HNSCC can develop from the mucosal epithelium of the oral cavity, the pharynx, the larynx or the nasal cavity. (**B**) Cellular and molecular landscape of the HNSCC tumor microenvironment. Schematic overview of major stromal and immune cell populations shaping HNSCC progression, including cancer-associated fibroblasts, endothelial cells, dendritic cells, T cells, natural killer cells, and tumor-associated macrophages. These populations communicate through chemokines, cytokines, and extracellular vesicles, collectively modulating tumor growth, immune surveillance, invasion, and therapy resistance. The molecular landscape of HNSCC is characterized by a low pH, hypoxia, elevated oxidative stress levels and a slightly increased temperature. Created in BioRender. Kummer, N. (2026) https://BioRender.com/hdzowjx (accessed on 9 February 2026).

Head and neck cancers (HNCs) are a group of aggressive and heterogeneous tumors that develop in several regions of the head and neck. In total, 90% of these cancers are classified as head and neck squamous cell carcinoma (HNSCC). They originate from the mucosal epithelium in the oral cavity, the pharynx, and the larynx, as well as the sinonasal cavity [1] (Figure 1A). With 890.000 new incidences and 450.000 deaths yearly (2018), HNSCC is the sixth most common cancer type worldwide. New cases are proposed to rise by up to 30% until 2030, possibly resulting in 1.08 million new cases yearly [2]. Risk factors for developing HNSCC include tobacco smoke, alcohol consumption and infection with viruses, including Epstein–Barr virus and human papilloma virus (HPV), primarily HPV-16, as well as exposure to environmental pollution, aging, an unhealthy diet lacking vegetables and poor oral hygiene [2]. Based on these different oncogenic noxae, HNSCCs can be categorized as HPV-positive or HPV-negative tumors. Despite many approaches for therapy, and though the overall survival rate has increased slightly over the last several decades, HNSCC is still associated with a high mortality rate and a five-year survival rate below 50%, as well as a relapse rate of around 50% [1,2]. This can be attributed to the lack of effective early detection or screening strategies, which frequently results in diagnosis at advanced disease stages [3]. Another reason accounting for these numbers is the limited efficiency of current treatment options. Treatment for HNSCCs is multimodal, mainly including surgical removal and a combination of chemotherapy and radiotherapy (=chemoradiotherapy) [2]. The standard-of-care treatment for HNSCCs consists of the chemotherapeutic drug cisplatin. Cisplatin is a very small, square planar molecule composed of one platinum atom, two amides and two chloride ions [4]. One of the main reasons for cisplatin-based treatment failure and one of the biggest clinical challenges is the occurrence of resistances, which can exist from the beginning or develop during the course of treatment [4,5]. Therapeutic resistance is driven by multiple mechanisms, comprising reduced intracellular drug accumulation, enhanced efflux, detoxification processes, and an increased capacity to tolerate DNA damage via activation of DNA damage response pathways and pro-survival signaling [6]. In order to overcome therapy resistance, these resistance mechanisms could be addressed with the described nanostructures. However, not all cisplatin resistance mechanisms are equally addressable by nanostructure-based approaches. Those involving pro-survival signaling pathways [7,8] and enhanced DNA damage response [9] mechanisms are particularly amenable, as nanostructures operate through targeted delivery and spatial control strategies that fundamentally differ from conventional combination therapies. Furthermore, beyond cisplatin, a broad spectrum of alternative active components may show potential in HNSCC, including natural bioactive compounds [10], small-molecule signaling modulators, regulatory RNAs [11], immunomodulatory proteins [12], and targeted protein binders, which aim to overcome resistance by interfering with oncogenic signaling, stress adaptation, DNA damage response, and tumor–immune interaction.

Considering the high rates of relapse, mortality, and therapy resistance, there is a clear need for novel and innovative therapeutic strategies that go beyond conventional chemotherapeutic approaches and instead target fundamental cellular properties of HNSCC.

### 1.2. Cellular and Molecular Landscape of Head and Neck Squamous Cell Carcinoma

The pathogenesis of HNSCC involves a multifaceted interplay of genetic, molecular, and cellular aberrations (Figure 1B) that influence each other and shape disease severity, progression and clinical outcome.

Genetic alterations include changes in expressions of genes like *TP53*, *NOTCH1*, *EGFR*, *CDKN2a*, *STAT3*, *CyclinD1* and *Rb* [13]. The most common genetic alteration in HNSCC is a mutation in the tumor suppressor gene *TP53*. It occurs in over 50% of all HPV-negative tumors and is associated with (radio)therapy resistance, the increased migration of cancer-associated fibroblasts (CAFs), elevated levels of reactive oxygen species (ROS) and metabolic reprograming of the tumor microenvironment leading to an enhanced acidic milieu, all contributing to tumor growth and progression [13]. While TP53 mutations represent loss-of-function alterations [14] that cannot be directly restored, nanostructure-based strategies can target survival pathways activated in p53-deficient cells, including enhanced DNA damage tolerance, mitotic stress adaptation, and reliance on anti-apoptotic regulators such as Survivin and STAT3.

Elevated ROS levels are a key characteristic of cancer [15]. Given the driving factors of HNSCC (mainly alcohol and tobacco), ROS levels are additionally increased in the tumor microenvironment (TME) of HNSCC (Figure 1B), which triggers many cellular pathways, supporting tumor growth and survival as well as therapy resistance [16]. Furthermore, elevated ROS levels in cancer cells can induce DNA damage in neighboring non-cancerous cells, additionally contributing to tumor progression [13].

Metabolic reprogramming is another key factor of the TME in HNSCC. Rapid tumor growth leads to an insufficient oxygen supply within the tumor, also referred to as hypoxia (Figure 1B). Hypoxic conditions in the tumor lead to the activation of hypoxia-inducible factor 1 (HIF-1). HIF-1 promotes metabolic reprogramming by increased glucose uptake and a shift from oxidative phosphorylation to aerobic glycolysis [17]. This leads to enhanced production of lactate and acidification of the TME. The acidic milieu within the TME of HNSCC furthermore promotes tumor growth and survival. The acidity is additionally associated with therapy resistance, as well as with the promotion of an immune-suppressive milieu through the polarization of macrophages into an M2 phenotype [18].

Cancer cells only account for approximately 30% of the cell population within a tumor [19]. The remaining cells are non-cancerous stroma cells, providing a supportive niche for tumor growth [20,21]. The TME comprises, besides cancer and stroma cells, various components, including CAFs and endothelial cells, as well as non-cellular compounds like the extracellular matrix (ECM) and signaling entities, such as chemo- and cytokines, hormones, paracrine factors, nutrients, and extracellular vesicles (EVs) [22] (Figure 1B). CAFs are a key component of the TME and promote many processes in HNSCC, including angiogenesis, epithelial–mesenchymal transition (EMT), immunomodulation, metabolic reprogramming, metastasis, therapy resistance and proliferation [23].

Another particularly important cell type in the TME, especially in HNSCC, is immune cells. Immune cells present in the TME include T- and B-lymphocytes, tumor-associated macrophages (M2 macrophages), natural killer cells (NK cells), myeloid-derived suppressor cells, dendritic cells, and neutrophils [20]. Those immune cells present in the TME display functions supporting the tumor in its growth rather than fighting it [24]. Increased growth mediated via immune cells is achieved via immune suppression within the tumor. For instance, regulatory T-cells (CD4^+^ T-cells), physiologically responsible for self-tolerance, can suppress the immune response within the tumor [25]. Tumor-associated M2 macrophages release, for example, high levels of Tumor Growth Factor ß (TGFß) and promote disease progression and metastasis, as well as therapy resistance [24,26]. Especially in HNSCC tumors, the immune microenvironment plays a very important role regarding the incidence, development, progression and prognosis of the disease. The TME in HNSCCs is one of the most immunosuppressive milieus among all solid tumors [2,21,27]. HNSCC tumors not only exhibit an immune-suppressive tumor site, but also enhance immune suppression systemically [24]. The immune-suppressive TME is based on the secretion of chemokines and cytokines from malignant cells, which block the function of effector immune cells, especially in later tumor stages [28].

Considering the highly immunosuppressive nature of the HNSCC tumor microenvironment, targeting immune-regulatory pathways emerges as a rational therapeutic strategy, for which nanostructures provide versatile and tunable platforms to selectively reshape antitumor immunity.

Overall, the limited treatment efficiency, the pronounced therapeutic resistance, the immunosuppressive tumor microenvironment, and the high relapse rates of HNSCC highlight the urgent need for innovative therapeutic strategies. In this context, the anatomical accessibility of mucosal tissues, combined with the dense stromal architecture, aberrant vasculature, and complex inflammatory milieu, renders HNSCC particularly suitable for the development and evaluation of stimulus-responsive and targeted nanostructured therapeutics with improved tissue penetration and microenvironment-adaptive functions.

## 2. Ancient Nanostructures as Biocompatible Platforms for Cancer Therapy

Ancient nanostructures, derived from naturally occurring minerals, biopolymers, and plant-based systems, represent some of the earliest nanoscale materials used in medicine. Long before the advent of modern nanotechnology, such materials were employed in traditional healing practices for wound care, inflammation control, detoxification, and infection management. Advances in materials science and nanomedicine have now enabled the rational redesign of these bio-inspired systems, positioning them as highly attractive platforms for cancer therapy, particularly in HNSCC. The appeal of ancient nanostructures lies in their intrinsic biocompatibility, biodegradability, and bioactivity, which contrast with many synthetic nanomaterials that may induce long-term toxicity or immune dysregulation. These properties are particularly advantageous in HNSCC, where treatment-associated morbidity and damage to healthy mucosal tissues remain major clinical challenges. Cancer treatment continues to face major challenges, including tumor recurrence, inadequate treatment monitoring, limited therapeutic efficacy, severe toxicity, and the emergence of multidrug resistance. Consequently, substantial advances in diagnostic and therapeutic technologies in this field remain difficult to achieve [29].

Long-term biodistribution, accumulation, and clearance are critical determinants of the clinical applicability of mineral-based nanoparticles, particularly in the anatomically complex and functionally sensitive head and neck region. Previous studies have shown that the in vivo fate of mineral-based nanoparticles is largely determined by their size, surface chemistry, crystallinity, and biodegradability [30]. Biodegradable mineral systems, such as calcium phosphate nanoparticles, allow clearance via endogenous metabolic pathways by gradually dissolving into physiologically relevant ions. Iron oxide nanoparticles, depending on their coating and size, can be metabolized via iron homeostasis mechanisms or cleared by the reticuloendothelial system. In contrast, non-biodegradable mineral nanoparticles, including some silica-based systems, raise legitimate concerns about long-term tissue retention and chronic toxicity, especially under conditions of repeated dosing [31]. Particularly in HNCs, localized or topical application strategies and image-guided application approaches have been proposed to limit systemic exposure and reduce off-target accumulation [32].

### 2.1. Mineral-Based Nanoparticles

Among the various platforms in nanomedicine, mineral-based nanoparticles stand out as the most historically significant and structurally resilient category of materials. Their extensive application in traditional medicine, combined with their physicochemical stability, has paved the way for their integration into contemporary oncological practices. At the nanoscale, systems derived from minerals present distinct advantages, including a high surface area, adjustable porosity, and inherent bioactivity, which allow them to serve as multifunctional carriers for both therapeutic and diagnostic agents. These characteristics are especially pertinent in cancer treatment, where the precise localization of drugs, controlled release, and reduction of off-target toxicity are critical clinical challenges that remain unresolved. As a result, mineral-based nanoparticles form an essential basis for the thoughtful design of bioactive nanocarriers aimed at treating head and neck cancers.

Mineral-derived nanostructures, including clay nanoparticles, silica, calcium phosphate (CaP), and iron oxide nanoparticles, constitute one of the oldest classes of nanomaterials used in medicine. At the nanoscale, these materials offer a high surface area, tunable porosity, and strong adsorption capacity, enabling efficient loading of chemotherapeutics, nucleic acids, and imaging agents.

Clay minerals frequently find application within the pharmaceutical sector, serving as either inert or activated components. The intricate chemical and physical properties of clay nanomaterials render these nanostructures ideal for the precise loading and release of various categories of therapeutic agents, including anticancer medications [33]. These minerals possess the remarkable ability to engage with the molecules of various therapeutic agents; additionally, they can also interact with diverse additive components, such as polymers, which may be utilized in the creation of nano-formulated medicines exhibiting optimal characteristics [34]. Numerous scholarly articles have been published on the application of nano-clays, such as MMT, HNT, and kaolinite, as nanocarriers for delivering anticancer medications [35]. These nano-formulations, derived from nano-clays, hold promise for the creation of tablets intended for oral administration, or they can be adeptly tailored for the development of systems designed for targeted delivery and controlled release of various anticancer agents [36]. Clay mineral nanostructures, exhibiting tunable physicochemical and morphological properties in various forms (flat, tubular, spherical, and fibrous), are natural nanomaterials and are emerging as systems with extraordinary potential for delivering various therapeutic agents to tumor sites. Their submicron dimensions, large specific surface areas, impressive adsorption capacities, chemical inertness, and multilayer structures ranging from 0.7 to 1 nm in thickness have generated significant interest in the scientific community as highly biocompatible nanosystems for cancer treatment. Among the most frequently investigated nano-clays in oncology are halloysite, bentonite, laponite, kaolinite, montmorillonite, and sepiolite. These multilayer minerals act as nanocarriers, enhancing stabilization, facilitating efficient transport, and enabling the sustained and controlled release of various anticancer agents (typically with a drug load of 1% to 10% by weight) [37].

Synthetic silica nanoparticles (SiNPs) have surfaced as adaptable nanomaterials with a wide array of applications spanning various fields. Their distinctive physicochemical characteristics, such as adjustable size, customizable surface chemistry, and remarkable stability, have garnered significant attention for a multitude of uses, from electronics and materials science to biomedicine [38]. Silica-based nanostructures exhibit notable features, such as a considerable internal surface area and pore volume, customizable pore dimensions, outstanding colloidal stability, and the capacity to alter the functionality of both the internal pore system and the external particle surface. As a result of these significant attributes, silica-based nanostructures have risen to prominence as a highly promising and versatile platform for biomedical applications [39]. SiNPs possess the remarkable ability to surmount these obstacles by proficiently transporting therapeutic agents to specific locations within the body. The application of SiNPs as carriers in the delivery of anticancer drugs has surfaced as a highly promising approach, attributed to their distinctive structural characteristics, biocompatibility, and adaptability. SiNPs amplify therapeutic effectiveness while minimizing systemic toxicity by providing benefits such as substantial drug-loading capacity, regulated release, and precise delivery [40]. However, debates regarding toxicity remain [38]. CaP nanoparticles exhibit remarkable biocompatibility and biodegradability, attributed to their chemical resemblance to human hard tissues, such as bones and teeth. These nanoparticles serve as effective carriers for a variety of biomolecules, including nucleic acids, proteins, peptides, antibodies, and drugs, which typically cannot penetrate cells to exert their biological effects. They can be loaded with cargo molecules through incorporation, in contrast to solid nanoparticles, as well as through surface functionalization. This not only provides protection against nucleases but also enables targeted delivery to specific cells. When these nanoparticles are functionalized with fluorescent dyes, they become suitable for imaging applications both in vitro and in vivo [41].

In order to address certain constraints associated with drug delivery methods, a variety of nanoparticle formulations have been introduced, such as biodegradable polymers, gold, iron oxide, liposomes, silica, dendrimers, and calcium phosphate-based mineral systems [42]. Biodegradable nanoparticles are typically favored for cancer treatment due to their predictable clearance pathways and mechanisms within the body, rendering them safer options for clinical use [43]. The ability for site-specific cellular entry is crucial for the bioactivity and bioavailability of the biomolecules being delivered. Research has indicated that the physical and chemical properties of CaP nanoparticles, such as size, charge, morphology, composition, and surface chemistry, are vital parameters that influence the internalization route of nanomedicines [44,45,46,47]. Additionally, other significant factors, including the dosage of bio-agents and their functionalities, are contingent upon the cellular entry pathway and the ultimate location within the cells [48]. Given that a cell contains numerous organelles, it is imperative for the delivery system to release its payload at the precise site to achieve the desired effect. Understanding how a cell directs its components to specific cellular compartments could enhance drug design, leading to improved tumor management [42]. Nanoparticles can be classified into two main types: hard and soft. Hard nanoparticles consist of inorganic substances and encompass quantum dots (QDs), noble metals, metal oxides, and lanthanide-based nanoparticles. Given their inorganic composition, concerns regarding toxicity and colloidal stability are prominent. However, a key benefit of inorganic nanoparticles lies in their capacity for physical and chemical surface modifications. Consequently, they are frequently modified with biocompatible materials, such as PEG, to enhance their safety and efficacy in vivo [32].

Metallic nanoparticles have inspired the notion that these particles could serve as drug delivery systems, owing to their remarkable chemical, physical, optical, and electronic properties, including high porosity, thermal stability, an open crystal structure, and the ability for controlled release [49,50]. Iron oxide nanoparticles have garnered significant attention in the realm of cancer treatment due to their unique properties, such as biocompatibility, customizable dimensions and surface chemistry, as well as their magnetic functionalities [51]. In cancer therapy, nanoparticles display a pivotal role in eliciting cellular reactions linked to host “danger” signals by engaging with particular receptors on phagocytic cells situated within the TME. Furthermore, they facilitate the activation of additional downstream antitumor immune pathways in the TME. Experimental studies utilizing iron oxide nanoparticles have highlighted localized antitumor immune modulation in preclinical cancer models, thereby reinforcing the advancement of this strategy aimed at fostering local antitumor immune responses [52]. Metallic nanoparticles are increasingly utilized as nanocarriers for drug delivery in HNC therapy. One approach involved the use of superparamagnetic nanoparticles through surface functionalization. This research was conducted by Zhang et al., who created an innovative drug delivery system utilizing magnetic nanoparticles specifically for HNCs. The system comprised biocompatible mesoporous Fe_3_O_4_ nanoparticles endowed with superparamagnetic characteristics, which were linked to polyacrylic acid (PAA). The therapeutic agent employed for HNC treatment was bleomycin (BLM), which could either be encapsulated within the mesoporous framework of the superparamagnetic nanoparticles or affixed to the PAA polymer’s surface via molecular crosslinkers. This polymer shell serves to diminish the natural clearance of superparamagnetic nanoparticles while controlling the drug’s release. These paramagnetic nanoparticles effectively transported BLM to the targeted region of the magnetic field within the tumor tissue, facilitating its gradual release and inducing apoptosis in tumor cells, all while minimizing the severe side effects of BLM on non-malignant cells and tissues. This novel strategy, characterized by its simplicity and lack of reliance on complex technologies, has demonstrated the capability to deliver the drug in a targeted fashion in vitro. It also inhibited tumor growth with therapeutic efficacy and reduced side effects in vivo, indicating significant potential for the use of nanomedicine in HNC treatment [53].

Metal nanoparticles, particularly gold nanoparticles (AuNPs), have attracted considerable interest as agents for photothermal therapy (PTT) owing to their exceptional optical characteristics, especially their pronounced absorption in the near-infrared (NIR) spectrum. In a study conducted by Zhang et al. [54], gold nanorods (GNRs) were synthesized and coated with EGFR monoclonal antibodies. Subsequently, previously treated laryngeal cancer cells were subjected to irradiation with an 808 nm NIR laser for a duration of 6 min, achieving a local average temperature of 50 °C, which led to apoptosis-mediated cell death. Similarly, cetuximab-coated GNRs, along with the human anti-EGFR antibody EGFR-hIgG1, were utilized in the HNSCC cell line CAL27. After a treatment period of 24 h, the cells were exposed to a 2 min dose of a continuous-wave (CW) diode laser operating at 1064 nm with a power density of 2 Wcm^−2^. It was observed that an increase in local temperature correlated with a significant reduction in tumor cells, suggesting that the combination of GNRs with targeted EGFR presents a promising strategy for effective PTT [55].

Since metal-based nanostructures are widely used in our daily lives due to their unique properties, their toxicity is of paramount importance. Studies have shown that the toxic effects of NPs are primarily determined by various factors such as physicochemical properties, dose, routes of exposure, and duration. While elucidating the precise mechanism of metal-based NP toxicity is challenging based on the current literature, recent studies have focused on oxidative stress as the underlying cause. Indeed, current in vitro and in vivo toxicity testing methods are primarily used to assess the acute and subacute toxicity of metal-based NPs, whereas nanotoxicity testing methods for chronic long-term NP exposure, which are crucial for predicting chronic toxicity in humans, are still lacking. Therefore, there is an urgent need to develop new powerful tools to evaluate and understand the mechanisms of metal-based NP toxicity. Many promising and effective strategies have been developed to design safer metal-based NPs to minimize NP toxicity. In conclusion, before a general consensus can be reached on the toxicity of nanoparticles, we need to comprehensively understand the toxicity of metal-based nanoparticles. In short, we believe that the convergence of related disciplines such as materials science, medicine, chemistry, and artificial intelligence will significantly advance the development of nanotoxicity research, thus making the use of metal-based nanoparticles safer in humans. [31].

### 2.2. Natural Biopolymer Nanostructures

While nanoparticles made from minerals offer structural stability and a high capacity for loading, natural biopolymer nanostructures present a complementary approach focused on biological adaptability and compatibility with tissues. Originating from naturally occurring macromolecules, these systems naturally replicate elements of the extracellular matrix and physiological environments, which enhances cellular interaction and biodegradation. When designed at the nanoscale, biopolymers provide exceptional flexibility regarding surface modification, responsiveness to stimuli, and kinetics of drug release. These characteristics effectively tackle the shortcomings of traditional cancer treatments, especially in anatomically delicate areas where it is crucial to maintain healthy tissue and mucosal integrity. As a result, biopolymer-based nanostructures have become highly adaptable platforms for developing safer and more effective cancer nanotherapeutics.

Natural biopolymers such as chitosan, silk fibroin, collagen, gelatin, and alginate have been used for centuries in regenerative medicine and wound healing. When engineered into nanoscale carriers, these materials demonstrate excellent biodegradability, mechanical tunability, and surface modifiability. Chitosan ranks as the second most abundant natural biopolymer following cellulose, and it is a modified natural cationic polysaccharide derived from the chemical deacetylation of chitin. The primary amino groups present in the polymer structure of chitosan confer positive charges to its surface. Due to its unique physical, chemical, and biological properties, it has emerged as a promising candidate for gene delivery within the gastrointestinal tract [50]. Regarding the selection criteria for an optimal biopolymer system for HNSCC, such as mucoadhesion and resistance to oral enzymes and microbiota, chitosan and its derivatives also offer advantageous properties for an application in the head and neck area.

In drug delivery systems that utilize chitosan, factors such as particle size, toxicity, thermal and chemical stability, as well as kinetics, are heavily influenced by the methods of preparation [56]. In the realm of cancer therapy, chitosan acts as a sophisticated drug carrier, significantly improving drug bioavailability and simultaneously minimizing adverse effects [57]. The anti-metastatic properties of chitosan stem from its mechanism that enhances penetration. A positively charged surface facilitates greater interaction with cell membranes, thereby boosting cell uptake and membrane permeability. The mucoadhesive qualities and permeability of chemotherapy agents can significantly influence drug absorption and the overall efficacy of the treatment.

Silk fibroin has garnered extensive application in the realm of biology, attributed to its exceptional biocompatibility, mechanical characteristics, biodegradability, and safety profile. In recent times, the advancement of silk fibroin as a drug carrier has accelerated, leading to significant breakthroughs in the treatment of cancer. The delivery system based on silk fibroin has demonstrated remarkable efficacy in eradicating tumor cells while minimizing side effects and preventing drug resistance [58]. Silk fibroin, a medical substance sanctioned by the US Food and Drug Administration (FDA), has found extensive application in postoperative suturing, tissue regeneration, and drug delivery systems [59]. Leveraging the remarkable mechanical properties along with exceptional biocompatibility and biodegradability, nanoparticles infused with drugs can be crafted utilizing silk fibroin to achieve a multitude of biological functions [60]. At present, there are no antitumor nanomedicines utilizing silk fibroin as a drug carrier available on the market. Currently, only albumin and silk fibroin share similar characteristics and classifications among the antitumor nanomedicines that have received marketing approval, offering valuable insights and a foundation for the future clinical application of silk fibroin in nanomedicine [58].

Collagen nanoparticles possess minimal antigenicity, an expansive surface area, non-toxic properties, and remarkable biocompatibility, rendering them highly promising for a variety of biomedical applications [61]. It has been acknowledged that cancer transcends the mere classification of a tumor cell disease; rather, it embodies a state of imbalance where stromal cells and the tumor microenvironment assume pivotal roles. The extracellular matrix (ECM), being the predominant element within the tumor microenvironment, possesses the ability to modulate the behaviors of tumor cells and maintain tissue tension homeostasis. Collagen serves as the foundational framework of the tumor microenvironment, influencing it by regulating ECM remodeling through collagen degradation and re-deposition, thereby facilitating tumor infiltration, angiogenesis, invasion, and migration. Historically, collagen was perceived as a passive barrier against tumor cells; however, it is now clear that collagen actively contributes to the advancement of tumor progression. Alterations in collagen within the tumor microenvironment emit biomechanical signals that are detected by both tumor and stromal cells, instigating a series of biological events [62]. Collagen-derived nanoparticles have been utilized in multiple applications as transport vehicles for pharmaceuticals, proteins, and genetic material. Collagen-derived nanoparticles exhibit advantageous physicochemical characteristics, including a diminutive size and an extensive surface area, which allow them to create colloidal solutions in aqueous environments and to persist within cells for extended durations [63].

Gelatin is a natural, biocompatible, biodegradable, bioactive, inexpensive, non-toxic, and multifunctional polypeptide structure. It possesses a polyampholytic character, containing both cationic and anionic groups along with hydrophobic components. This substance can be obtained through acid, alkali, or enzymatic hydrolysis of collagen. Commercially, gelatin is available in both cationic (gelatin type A, isoelectric point (pI) 7–9) and anionic (gelatin type B, pI 4.8–5) forms, requiring no additional functional processing [50]. Among the numerous biomaterials explored for drug delivery nanosystems, gelatin, a derivative of hydrolyzed collagen, emerges as a highly versatile polymer approved by the FDA, showcasing significant potential for anticancer applications. The natural abundance, affordability, and polyampholytic characteristics of gelatin facilitate easy chemical modification and crosslinking, rendering it particularly appropriate for sustained and controlled drug release. Gelatin-based nanoparticles (GNPs) present the benefit of being responsive to stimuli that are specific to tumors, including alterations in pH or enzymatic activity. This characteristic facilitates targeted, site-specific drug release, thereby minimizing renal clearance and systemic adverse effects. The versatile characteristics of gelatin as a biomaterial enable it to experience both structural and chemical changes in reaction to diverse environmental stimuli, thereby augmenting its applicability in targeted drug delivery. Importantly, gelatin exhibits a high sensitivity to variations in pH, temperature, and enzymatic activity, positioning it as a prime candidate for drug release systems that respond to stimuli [64].

Alginate is an acidic colloidal polysaccharide found in the ocean, functioning as both a biopolymer and a polyelectrolyte. It is known for its biocompatibility, non-toxicity, non-immunogenicity, and biodegradability. Numerous studies have confirmed the potential of alginate-based platforms as effective carriers, particularly for drug delivery in cancer therapy [65]. Alginate has demonstrated significant promise as a biomaterial for a range of biomedical applications, particularly in the areas of wound healing, drug delivery, in vitro cell culture, and tissue engineering. Both in vitro and in vivo studies indicate the safety of chemically diverse types of alginate nanoparticles [66]. Nanoparticles based on alginate, an anionic polysaccharide that is commonly found in the cell walls of brown algae and forms a viscous gum upon contact with water, have become one of the most thoroughly studied biomaterials for drug delivery and targeting various administration routes. The benefits of these nanoparticles include not only their diverse physicochemical properties, which facilitate chemical modifications for targeted delivery, but also their biocompatibility, biodegradability, and mucoadhesive characteristics.

When studies in the literature are examined, it can be found that chitosan-decorated polycaprolactone microparticles (CS-PCL MPs) enabled high 5-fluorouracil (5-FU) loading (~39%) and sustained release for up to 96 h. The system significantly inhibited human HNSCC cell growth and colony formation while sparing normal fibroblasts, through induction of autophagy and apoptosis. A higher chitosan (CS) content further enhanced tumor growth and motility suppression, likely due to the positive charge-mediated selective cytotoxicity of CS. Overall, CS-PCL MPs represent a promising drug delivery platform to improve 5-FU efficacy in HNSCC [67].

In the literature, silk fibroin (SF) also stands out as a very promising candidate. SF-based nano-drug carriers were developed using Arg-Gly-Asp–SF–polylactic acid (RSA) to co-encapsulate doxorubicin (Dox) and atovaquone (Ato), forming micelle-like nanoparticles (RSA-Dox-Ato NPs). RGD decoration enhanced tumor targeting, while Ato, a mitochondrial complex III inhibitor, alleviated tumor hypoxia and improved chemotherapeutic efficacy. The carrier showed minimal intrinsic cytotoxicity, whereas RSA-Dox-Ato NPs significantly increased tumor cell inhibition and intracellular reactive oxygen species (ROS) levels, indicating hypoxia reversal. Overall, SF-based targeted nanoparticles effectively enhanced chemotherapy and suppressed tumor growth, highlighting their promise as a biocompatible drug delivery system [68].

### 2.3. Plant-Derived Nanovesicles (PDNVs)

Beyond mineral and polymeric platforms, plant-derived nanovesicles signify a biologically advanced evolution of traditional plant-based therapeutic systems. In contrast to synthetic or semi-synthetic nanocarriers, PDNVs are naturally occurring lipid bilayer structures that are rich in bioactive molecules, facilitating intercellular communication across different species. Their natural biocompatibility, minimal immunogenicity, and inherent biological activity set them apart as new nanotherapeutic agents. Importantly, PDNVs have the ability to influence the TME while also acting as vehicles for therapeutic substances. These features make PDNVs strong contenders for overcoming treatment resistance and toxicity, presenting a biologically cohesive strategy for cancer treatment.

Plants naturally secrete nanoscale vesicles that encapsulate bioactive lipids, polyphenols, flavonoids, proteins, and regulatory RNAs. Recent evidence demonstrates that plant-derived nanovesicles are readily internalized by mammalian cells and exert potent anti-inflammatory and anticancer effects.

Plant extracellular vesicles (PEVs) were initially observed through transmission electron microscopy (TEM) in the 1960s. Plant-derived vesicles are membrane-bound structures originating from plant cells and are responsible for numerous physiological and pathological functions. In recent years, researchers have intensified their investigations into plant-derived vesicles to clarify the potential for wider applications of more biocompatible materials in cancer therapy, as opposed to the synthetic medications that are currently prevalent. For therapeutic purposes, plant-derived vesicles must be taken up by target cells without inducing adverse side effects or systemic toxicity in healthy cells. The current literature indicates that PDVs demonstrate promising potential in cancer treatment through two mechanisms: (I) their natural anticancer properties as biologically active phytomedicines and (II) transporting active agents to targeted tumors as drug-delivery vesicles [69]. Plant-derived nanovesicles are naturally occurring bioactive lipid bilayer structures that encompass proteins, lipids, ribonucleic acid, and metabolites. They have demonstrated the ability to enhance cell growth, migration, and differentiation into various tissue types. With their immunomodulatory, microbiota-regulating, antioxidant, and anti-aging properties, plant-derived nanovesicles are instrumental in countering external stimuli and promoting tissue repair [70]. The anticancer effects of various types of PDVs have been studied and found to engage in multiple mechanisms. Generally, PDVs are known to suppress the proliferation of cancer cells while facilitating their death, all without causing adverse effects on non-cancerous cells. Citrus-derived nanovesicles have been shown to reduce the viability of tumor cell lines such as A549, SW480, and LAMA84 in a manner that is dependent on both dose and time. In contrast, other normal cell lines (HS5, HUVEC, and PBMC) subjected to the same treatment conditions exhibited no indications of damage. Subsequent analyses indicated that these vesicles induced cancer cell death through the activation of TRAIL-mediated apoptosis, a finding that was further validated in the in vivo LAMA84 xenograft model [71]. A multitude of research studies have demonstrated that plant-derived exosome-like nanoparticles serve as mediators of communication between cells and that plant-derived exosome-like nanoparticles may play a role in the therapeutic management of various diseases. In addition to inducing apoptosis and causing cell cycle arrest, PDVs also seem to influence the tumor microenvironment. Nanoparticles derived from ginseng have been demonstrated to modify macrophage polarization both in vitro and in vivo through a TLR4-MyD88-dependent pathway, ultimately leading to the inhibition of tumor growth. Furthermore, these particles were confirmed to be biocompatible, exhibiting no negative effects on healthy cells or mouse models. Notably, the removal of proteins from these vesicles diminished their uptake by ovarian cancer cells and reduced the upregulation of M1-related surface markers when compared to normal vesicles, thereby underscoring the crucial role of proteins in the bioactivity of ginseng plant-derived exosome-like nanovesicles [72]. Additionally, ginseng plant-derived exosome-like nanovesicles were assessed for their potential in combination with the programmed cell death protein-1 monoclonal antibody. This combination demonstrated the capacity to modify the cold tumor microenvironment and subsequently elicit sustained systemic antitumor immunity in vivo [73]. In summary, PDNVs embody a novel and adaptable category of therapeutics, showcasing remarkable potential within the realms of life sciences, pharmacology, and medicine. Their biostability and cost-effectiveness render them highly promising candidates for diverse therapeutic applications. Ongoing research and interdisciplinary collaborations are essential for optimizing the utilization of PDNVs, enhancing clinical practices, and fostering the development of innovative and personalized treatment options.

Given the encouraging anticancer effects demonstrated in both in vitro and in vivo studies, PDNV-based therapy is regarded as a promising strategy for cancer treatment, with several early-stage clinical trials already completed or currently underway [74].

A recently completed clinical study evaluated grape-derived vesicles for the prevention of chemotherapy-induced oral mucositis in HNC, while also assessing their effects on cytokine profiles, immune responses to tumor exome antigens, and patient metabolic and molecular markers. In parallel, an ongoing clinical trial is investigating PDVs as carriers to enhance curcumin delivery and bioavailability in normal and colon cancer tissues. Although both studies indicate that PDV-based therapies are clinically safe, their therapeutic efficacy has not yet been reported and remains under evaluation [69].

In summary, plant-derived nanovesicles are gaining increasing attention as biocompatible and bioactive drug delivery systems; however, their transition to clinical applications is constrained by several key challenges. In particular, significant compositional heterogeneity stemming from differences in plant species, tissue origin, growing conditions, and isolation protocols leads to batch-by-batch variability in lipid, protein, and RNA content, compromising reproducibility and mechanistic interpretation. Furthermore, commonly used isolation techniques are largely labor-intensive and difficult to scale up, limiting the development of robust, good manufacturing-practice-compliant production lines. Additionally, plant-derived nanovesicles lack well-defined regulatory classification and standardized characterization criteria, making quality control, safety assessment, and regulatory approval challenging. Collectively, these issues underscore the need for compatible manufacturing and evaluation frameworks to reliably translate plant-derived nanovesicles into clinical applications [75].

## 3. Emerging Nanostructures for Targeting Head and Neck Cancer

**Figure 2:**
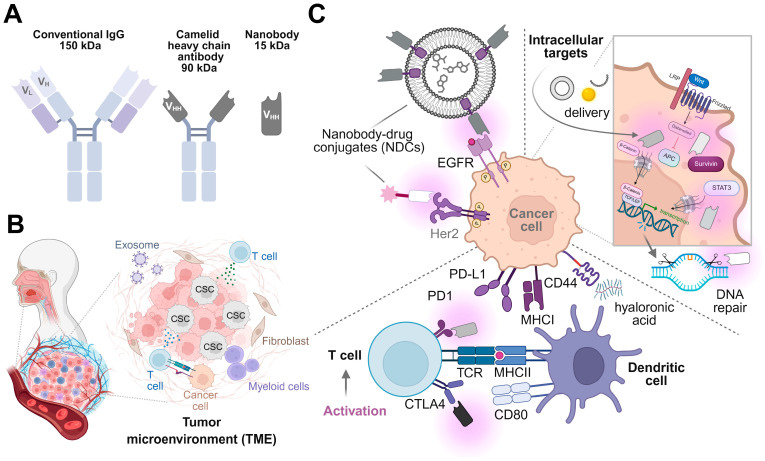
Schematic overview of nanobody-based strategies in head and neck squamous cell carcinoma (HNSCC). (**A**) Comparison of conventional antibodies, camelid heavy-chain antibodies, and nanobodies. (**B**) Representation of the tumor microenvironment, including cancer cells, cancer stem cells, immune cells, stromal cells, and exosomes. (**C**) Nanobody–drug conjugates and nanobody-based platforms for targeted delivery, immune regulation, and modulation of intracellular signaling and DNA repair pathways. Created in BioRender. Knauer, S.K. (2026) https://BioRender.com/hy7o5ps (accessed on 9 February 2026).

Head and neck squamous cell carcinoma continues to pose substantial therapeutic challenges, driven by marked tumor heterogeneity, limited drug penetration and a high capacity for adaptive resistance [76]. While nanotechnology has long promised improvements in drug delivery and targeting, many nanosystems struggle to translate molecular specificity into robust clinical benefit [77]. Nanobodies, single-domain antigen-binding fragments derived from camelid heavy-chain antibodies, occupy an unusual and productive position at the interface of biology and nanotechnology (Figure 2A) [78,79]. Their small size, structural simplicity, and adaptability allow them to function not merely as antibody surrogates but as biologically encoded nanostructures capable of addressing several limitations that define HNSCC therapy (Figure 2B) [80,81].

### 3.1. Nanobodies as Precision Therapeutic Nanostructures in HNSCC

At approximately 12 to 15 kDa, nanobodies are an order of magnitude smaller than conventional immunoglobulins [78,82] (Figure 2A). This difference is not cosmetic. In HNSCC, where irregular vascularization, dense stromal compartments, and hypoxic tumor regions impede homogeneous drug distribution (Figure 2B), molecular size becomes a decisive parameter [83,84,85]. Nanobodies diffuse more efficiently through tumor tissue and retain binding activity under conditions that compromise larger proteins [79]. Their single-domain architecture confers exceptional stability across a range of pH and redox environments, properties that are particularly relevant in advanced or treatment-resistant lesions. From a conceptual perspective, nanobodies can be regarded as evolutionarily optimized nanoscale recognition units whose intrinsic physicochemical properties align well with the constraints imposed by solid tumors of the head and neck [86]. The target landscape of HNSCC further underscores the suitability of nanobody-based strategies [87]. Surface receptors such as EGFR, PD-L1, HER2 (in selected subgroups), and CD44 variants remain clinically relevant but incompletely addressed by existing antibody therapeutics (Figure 2C) [87,88,89,90,91,92]. Resistance to EGFR-directed treatments, for example, frequently arises through altered receptor conformation, compensatory signaling, or changes in receptor trafficking rather than simple loss of expression [93,94,95,96]. Nanobodies can engage epitopes that are inaccessible to full-length antibodies and may distinguish between functional receptor states, offering opportunities to bypass some of these resistance mechanisms [97,98]. Their rapid tissue penetration and fast systemic clearance also support applications in imaging and short-acting therapeutic interventions, which are particularly attractive in anatomically sensitive regions [99,100,101]. While the small size of nanobodies promotes deep tumor penetration, rapid renal clearance can limit systemic exposure. Importantly, this pharmacokinetic constraint can be addressed without compromising targeting specificity through several established strategies, including fusion to albumin-binding modules, multivalent or biparatopic nanobody formats that enhance tumor retention via avidity effects, and site-specific half-life extension approaches such as PEGylation or PASylation distal to the antigen-binding interface. In addition, conjugation to therapeutic or diagnostic payloads and nanomaterials can modulate biodistribution while preserving antigen selectivity. Notably, the anatomical accessibility of HNSCC further enables loco-regional delivery strategies that mitigate systemic clearance altogether. Collectively, these approaches decouple pharmacokinetics from targeting fidelity and support the clinical translation of nanobody-based modalities in HNSCC.

Importantly, the relevance of nanobodies in HNSCC extends beyond surface antigens. Many of the pathways driving tumor progression and therapy resistance converge on intracellular proteins that remain poorly druggable using classical approaches. Factors such as Survivin [7], STAT3 [102], components of the Wnt [103,104] and DNA damage response pathways [9,105], and regulators of nucleocytoplasmic transport [106,107,108,109,110] exert their oncogenic functions through protein–protein interactions and dynamic subcellular localization rather than enzymatic activity [111,112,113]. Nanobodies can be engineered for intracellular expression or delivery, where they act as highly specific binders capable of modulating protein conformation, localization, or interaction networks [114,115,116]. This intracellular reach represents a qualitative expansion of the therapeutic space in HNSCC, rather than a marginal optimization of existing strategies. The modularity of nanobodies further enables their integration into functional nano-platforms tailored to the specific demands of head and neck cancer treatment [117]. Intracellular nanobody targeting is indeed a promising strategy, but its in vivo implementation remains constrained by a combination of biological and technological barriers. Biologically, nanobodies are inherently membrane-impermeable and typically enter cells via endocytosis, which frequently results in endosomal sequestration and lysosomal degradation rather than functional cytosolic availability. Limited endosomal escape represents a major bottleneck, while additional challenges include maintaining cytosolic stability in a proteasome-rich environment and, for nuclear targets, achieving efficient nuclear import within a sufficient intracellular residence time. Importantly, recent work demonstrates that some of these barriers can be overcome through optimized delivery systems. In particular, lipid-mediated intracellular delivery of recombinant bioPROTACs has been shown to enable efficient cytosolic access and rapid degradation of otherwise undruggable intracellular targets in vivo [118]. These findings provide a compelling proof of concept that nanobody-based intracellular modalities can be functionally effective when coupled to delivery platforms that promote endosomal escape. However, such approaches currently rely on complex lipid or carrier formulations whose efficiency, biodistribution, and tolerability remain highly context- and formulation-dependent. As a result, while delivery technologies are rapidly advancing, the lack of broadly applicable, target-agnostic systems continues to represent the principal technological barrier to widespread in vivo translation of intracellular nanobody therapeutics, including in HNSCC.

Nanobody–drug conjugates offer an alternative to conventional antibody–drug conjugates, combining molecular precision with improved tissue penetration and potentially more uniform intratumoral payload distribution [119]. In parallel, nanobodies are increasingly used as targeting elements for synthetic nanoparticles, including lipid-based carriers, polymer systems, and inorganic nanomaterials [101,120,121]. In these hybrid constructs, nanobodies provide the molecular address, while the nanoparticle core governs pharmacokinetics, release kinetics, or imaging properties [122]. Such division of labor is particularly advantageous in HNSCC, where local accessibility and multimodal treatment regimens create opportunities for combining systemic targeting with spatially confined therapeutic activation [86,123]. A particularly promising and still emerging application lies in nanobody-based targeted protein degradation [82,124,125]. By coupling intracellular nanobodies to E3 ubiquitin ligase recruitment domains, bioPROTAC systems enable selective elimination of oncogenic proteins rather than transient inhibition [126,127,128]. This approach is well suited to proteins that function as scaffolds or regulatory hubs, including Survivin, which plays central roles in mitosis, stress adaptation, and DNA damage tolerance in HNSCC [129,130]. Targeted degradation introduces a catalytic mode of action that can overcome high protein abundance and reduce the likelihood of adaptive resistance, offering a fundamentally different strategy for pathway intervention [131].

Beyond direct tumor cell targeting, nanobodies also provide tools for probing and modulating the tumor microenvironment (Figure 2B) [85]. Their favorable size and kinetics support applications in immune modulation, stromal targeting, and high-resolution molecular imaging. Radiolabeled nanobodies, in particular, enable rapid and high-contrast visualization of tumor lesions and margins, which is of practical relevance for surgical planning and adaptive radiotherapy in HNC [100,132]. Bispecific nanobody formats further allow the simultaneous engagement of tumor antigens and immune effectors, creating opportunities for spatially restricted immune activation [81,133,134]. From a translational perspective, challenges such as short serum half-life and efficient intracellular delivery remain. Nanobodies’ small size leads to rapid renal clearance, necessitating half-life extension strategies (e.g., Fc fusion and albumin binding), while intracellular targeting requires additional engineering approaches such as cell-penetrating peptides, nanoparticles, or vector-based expression to achieve cytosolic or nuclear access [78,115,135]. However, these limitations are increasingly being addressed through rational engineering and through integration with advanced nanostructured carriers that provide protection, controlled release, or stimulus-responsiveness. Strategies include nanobody fusion to albumin-binding domains or Fc fragments to extend serum half-life, incorporation of cell-penetrating peptides for intracellular access, and conjugation to nanoparticles or hydrogels that allow tumor-targeted accumulation and stimulus-triggered payload release [115,132,135,136,137,138]. In this context, nanobodies serve less as standalone therapeutics and more as programmable recognition modules within larger, multifunctional systems. In summary, nanobodies illustrate how biologically derived nanostructures can be repurposed to meet the specific biological and clinical constraints of HNC. Their capacity to access dense tumor tissue, engage unconventional targets, and integrate seamlessly into complex nano-platforms positions them as central components of next-generation strategies aimed at overcoming resistance and improving therapeutic precision in HNSCC (Figure 2, Table 1).

### 3.2. Engineered Exosomes as Potential Modulators of Cellular Signaling Pathways in HNSCC

**Figure 3:**
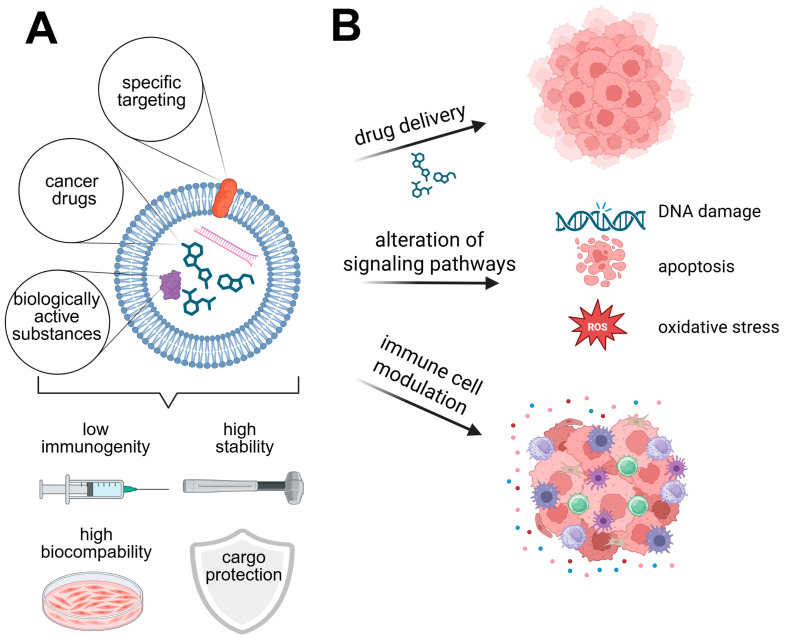
Exosome-based drug delivery strategies in cancer therapy. (**A**) Exosomes can encapsulate anticancer drugs and mediate passive delivery, while surface-associated proteins or engineered ligands enable specific targeting of tumor cells, thereby enhancing therapeutic efficacy and selectivity. (**A**,**B**) Tailoring the molecular cargo of exosomes enables the alteration of downstream signaling pathways as well as the modulation of immune cells in the TME of HNSCC. (**A**) Key advantages of engineered exosomes as nanocarriers include low immunogenicity, high biocompatibility, enhanced structural stability, and effective cargo protection. Created in BioRender. Kummer, N. (2026) https://BioRender.com/hdzowjx (accessed on 9 February 2026).

Exosomes are nanoscale extracellular vesicles originating from the endosomal pathway that function as active mediators of intercellular communication. By transferring bioactive cargo, such as proteins, lipids, and nucleic acids to distant and adjacent cells, they directly modulate gene expression, signaling pathways, and cell fate decisions in recipient cells [139]. Thereby, they are involved in many key physiological processes of the recipient cells, including homeostasis, the regulation of immune responses, and proliferation, as well as differentiation [139,140]. Beyond their significant role in normal tissue physiology, exosomes are additionally involved in pathological conditions [141], for instance, by being a part of the tumor microenvironment (Figure 1B), where they contribute to tumor progression, therapy resistance, and immune modulation [139,142]. In the tumor microenvironment exosome-mediated communication reshapes cellular behavior by influencing stress responses, survival signaling, and phenotypic plasticity of both tumor and stromal cells. For instance, exosomes within the tumor microenvironment can carry pro-survival proteins, including Survivin, which induces apoptosis inhibition in cancer cells, leading to their survival [143]. Additionally, molecules such as ΔNp73 that inhibit the function of tumor suppressors, including p53, can be transported, leading to cancer cell survival [144]. This shows that, despite their small size of around 30–200 nm [139], exosomes play important parts in many essential cellular processes by carrying molecular cargo and thereby hold a lot of potential in clinical engineering applications. Given their natural function as “cargo-carriers” and thereby cell biology regulators, they are highly promising entities, both as nanocarriers for drugs but also as biological agents influencing behavior and cellular processes. Furthermore, differing from synthetic nanomaterials (i.e., liposomes and polymer-based nanoparticles), exosomes exhibit low immunogenicity [145]. They furthermore consist of a thick lipid bilayer (schematically shown in Figure 3A), which is rich in, e.g., cholesterol [139], providing a robust protective barrier that preserves the integrity of their internal cargo [146]. Their membrane composition furthermore enables prolonged circulation and efficient accumulation within tumors, addressing delivery challenges common in HNSCC treatment [147]. All these naturally occurring functions, such as their biocompatibility, stability, and high capacity, make them ideal candidates for therapeutic delivery applications in HNSCC (Figure 3A). Engineering EVs to carry anticancer agents has opened new routes for targeted cancer therapy, potentially overcoming challenges of drug resistance and off-target effects [148].

To achieve precise modulation of cellular processes in recipient cells, the inherent biological properties of exosomes can be exploited and engineered using diverse strategies. Their molecular cargo can be tailored toward manifold defined biological functions, depending on the desired downstream application. Both therapeutic agents, such as anticancer drugs, and biologically active molecules, including proteins and nucleic acids, can be specifically incorporated into exosomes using endogenous or exogenous loading strategies.

Endogenous cargo loading occurs prior to exosome isolation and is achieved through genetic engineering of exosome donor cells. In this approach, donor cells are transfected with plasmids encoding the desired protein or RNA, resulting in transient expression of the exogenous construct. During exosome biogenesis, these expressed molecules can be packaged into exosomes and subsequently transferred to recipient cells following exosome isolation and exosome uptake. This strategy enables physiological incorporation of regulatory molecules into exosomes and has been widely used to generate exosomes with defined biological activity [149]. One illustrative example is transfection of HEK293T donor cells with a plasmid encoding for specific proteins, which were in turn packaged into the exosomes by the engineered cells [150].

In contrast, exogenous cargo loading is performed after exosome isolation and enables direct incorporation of defined agents into purified vesicles. Common post-isolation loading methods include electroporation, sonication, freeze–thaw cycles, extrusion, and incubation of cargo with exosomes [151]. These approaches are particularly useful for incorporating molecules that cannot be efficiently introduced into donor cells by transfection.

Regardless of the loading strategy employed, engineered exosomes are efficiently taken up by recipient cells, where their cargo can modulate signaling pathways, gene expression programs, and cellular behavior (Figure 3B). Through these mechanisms, cargo-loaded exosomes can actively reshape tumor and stromal cell responses within the HNSCC tumor microenvironment.

Cellular entry of exosomes is highly regulated and occurs through different mechanisms, for instance, membrane fusion and cargo release into the cytoplasm or phagocytosis. One described mechanism is the exosomal ligand–cell receptor-based uptake which triggers intracellular pathways in the cells [139] and offers intrinsic properties for the engineering of exosomes to specifically target desired cells.

For the specific targeting of (cancer) cells, the exosome surface can be engineered. These strategies enable the display of targeting ligands or receptors on the exosome surface, thereby enabling cell-specific recognition, uptake of exosome cargo, and therapeutic efficacy in targeted drug delivery (Figure 3A) [152]. For example, one study reported the generation of engineered exosomes through donor cell modification to present HER2-specific targeting motifs on the exosomal surface while simultaneously incorporating therapeutic cargo, resulting in selective delivery of cargo to HER2-positive cancer cells only [11]. In HNSCC, EGFR is highly overexpressed in most of the patients and drives key signaling pathways involved in tumor proliferation, survival, and therapy resistance [88]. In this context, engineered exosomes functionalized with EGFR-binding peptides such as GE11 enable selective recognition and uptake by EGFR-positive HNSCC cells, thereby enhancing the cell-type-specific delivery of therapeutic cargo [153]. Engineering exosomes to specifically target cancer cells expressing specific receptors minimizes their off-target effect and delivery of molecules to undesired cells.

Accordingly, exosomes can be engineered to display a wide range of targeting ligands, enabling selective interactions not only with tumor cells but also with other cell types, including immune cell populations and CAFs. One study reported that engineering extracellular vesicles with CAF-targeting surface modifications and loading them with miR-138-5p and pirfenidone selectively suppressed TGF-β signaling and collagen production in CAFs, thereby reversing their pro-tumorigenic phenotype in a pancreatic tumor model [154]. Although direct evidence for engineered exosome-mediated CAF reprogramming in HNSCC is currently lacking, these findings highlight the strong potential of such strategies to be translated to HNSCC, given the prominent role of CAFs in shaping its stromal architecture, therapy resistance, and tumor progression [23]. Furthermore, engineered exosomes enable modulation in immune signaling within the tumor microenvironment. The TME in HNSCC is one of the most immunosuppressive milieus among all solid tumors [2,21,27], consisting of many immune cells exhibiting immune-suppressive functions, such as regulatory T-cells, tumor-associated B-cells, and dendritic cells, as well as tumor-associated macrophages (Figure 1B) [24]. The possibility of altering this immune-suppressive milieu offers great potential in the treatment of HNSCC. For example, a recent study presented engineered exosomes programmed to co-display PD-1 and the Wnt receptor FZD8 on their surface, which enabled simultaneous blockade of PD-L1 checkpoint signaling and Wnt7b-driven immunosuppressive pathways. This led to enhanced cytotoxic CD8^+^ T-cell activation and reprogramming of the tumor immune microenvironment in checkpoint therapy-resistant models [12], which could potentially be applied in HNSCC models as well.

Although less extensively studied, engineered exosomes may also modulate additional cellular pathways, as they are intrinsically involved in a wide range of cellular processes of importance in HNSCC, including DNA damage response signaling, ROS generation, senescence and stemness. Exosomes were shown to actively influence DNA damage response in recipient cells. For instance, exosomes derived from mesenchymal stem cells can attenuate cytotoxic effects of common chemotherapeutic drugs like cisplatin and bleomycin in HeLa cancer cells, as displayed by decreased γH2AX expression. Simultaneously, intracellular ROS levels were decreased in exosome-treated cells [155]. Exosomes have significant roles in oxidative stress signaling. Inhibition of exosome release was shown to significantly increase intracellular oxidative stress, underscoring their essential role in maintaining cellular redox homeostasis [156]. Furthermore, mesenchymal stem cell-derived exosomes exhibit strong antioxidant properties [157], highlighting their potential engineering application in HNSCC, which is marked by chronically elevated oxidative stress, driven by metabolic reprogramming, inflammatory signaling, and hypoxic conditions (Figure 1B). This redox imbalance profoundly influences DNA damage accumulation, stress adaptation, and cell fate decisions in both tumor and stromal cells [16]. Additionally, exosome-mediated signaling contributes to the spread and maintenance of cellular senescence by disseminating senescence-associated factors that reshape the tissue microenvironment [158].

Building on the central role of exosomes in cancer-associated cellular signaling pathways, their rational engineering represents a promising strategy to overcome current therapeutic limitations in HNSCC. Engineered exosomes may modulate DNA damage response signaling pathways that otherwise promote tumor cell survival following therapy. Moreover, exosome-mediated regulation of cellular senescence, a key contributor to therapy resistance, could be exploited to improve treatment efficacy. In addition, exosomes may function as scavengers of reactive oxygen species, thereby further enhancing antitumor effects.

Beyond influencing cellular signaling, exosomes can also function as active carriers of antitumor reagents. A study showed that engineered exosomes loaded with siRNA were able to efficiently deliver their cargo to oral squamous cell carcinoma cells, leading to targeted silencing of the *LCP1* gene and pronounced antitumor effects in vitro and in vivo [159]. This highlighted the potential of exosomes as carriers of bioactive molecules to influence cellular gene expression.

In addition to RNAi components, exosomes can also be exploited for the delivery of chemotherapeutic drugs. Successful loading and release of doxorubicin has been shown in several studies already [10,160]. Not only was successful loading and release shown, but the drug accumulation was also shown to be much higher, while cytotoxicity to non-cancerous cells was reduced, making exosomes an ideal potential drug deliverer [161,162]. Of great importance for HNSCC is the packaging of cisplatin, the standard-of care chemotherapeutic agent applied in HNSCC. Although the packaging of cisplatin is not the same as that described for doxorubicin, for example, successful packaging and release of cisplatin into exosomes has also already been achieved. This study revealed decreased cell proliferation as well as apoptosis in target cells, followed by a suppression of the cancer-relevant protein Survivin [163]. Other chemotherapeutics like Paclitaxel, as well as non-chemotherapeutics like curcumin and celasterol, could be successfully packaged into exosomes, highlighting their huge potential as nanocarriers for anticancer treatments and a variety of other compounds (Figure 3, Table 1) [10].

### 3.3. DNA Origami Nanostructures for Drug Delivery as Well as Ligand-Specific Targeting in HNSCC

**Figure 4:**
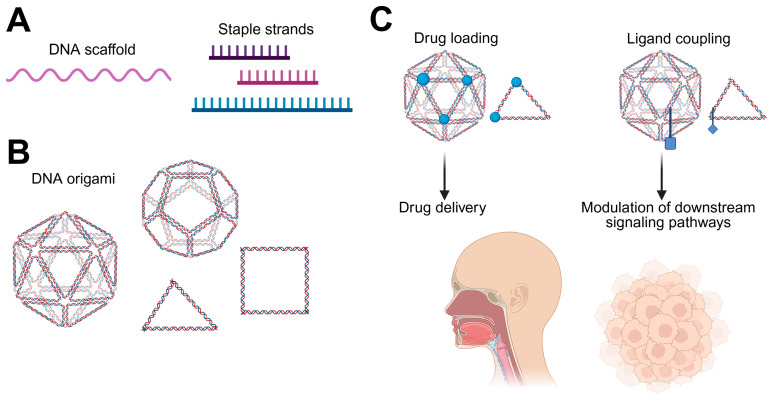
Schematic illustration of DNA origami-based drug delivery for head and neck squamous cell carcinoma (HNSCC). (**A**,**B**) A long single-stranded DNA scaffold is folded by complementary staple strands to form defined DNA origami nanostructures of different geometries. (**C**) These nanostructures can be loaded with therapeutic agents and targeting ligands and subsequently used for targeted drug delivery and modulation of downstream signaling pathways. Upon administration, drug-loaded DNA origami nanocarriers facilitate the transport and release of the cargo to tumor tissue in head and neck squamous cell carcinoma. Created in BioRender. Kummer, N. (2026) https://BioRender.com/hdzowjx (accessed on 9 February 2026).

DNA origami refers to the programmable self-assembly of DNA into precisely defined two-and three-dimensional nanostructures (Figure 4A,B) and was first described in 2006 by Rothemund, with subsequent expansion for three-dimensional folding three years later [164,165]. This technique relies on folding a long single-stranded DNA scaffold into predefined nanoscale shapes using numerous short staple strands through sequence-specific base pairing (Figure 4A) [166]. Due to their high structural precision and programmability (Figure 4B), DNA origami nanostructures provide an ideal nano-platform for the controlled incorporation and spatial organization of therapeutic agents, genes as well as targeting ligands [167] (Figure 4C). Specific loading into DNA origami is enabled by the high density of DNA double helices, which allows for non-covalent intercalation or hybridization of small-molecule drugs, as well as by covalent attachment of drugs or ligands to defined chemically modified DNA strands, which provide defined sites for this covalent conjugation. This allows for very precise control over drugs and positioning, as well as stability and drug release [166,168,169]. Although DNA origami structures in the context of HNSCC have barely been described in the literature, their application appears highly promising for future therapeutic strategies. Cases of HNSCC often face the challenges of a high rate of resistance to the standard-of-care chemotherapeutic agent cisplatin [170] as well as a high relapse rate [2], highlighting once again the need for advanced and more effective treatment methods. In addition, conventional chemotherapeutic drugs often suffer from unspecific targeting and systemic cytotoxicity, which significantly limits their therapeutic efficiency [169]. The application of nucleic nanostructures, such as DNA origami for drug delivery, offers a great potential solution to overcome these limitations. In cancer therapy, DNA origami-based drug delivery systems have already demonstrated promising results. Several studies have reported the successful loading and delivery of anticancer drugs, with most investigations focusing on doxorubicin, although alternative therapeutic agents have also been explored. For example, doxorubicin loading into triangular DNA origami nanostructures and subsequent efficient delivery into ovarian cancer cells has been demonstrated [171]. Furthermore, in vivo studies have shown that DNA origami can function as an effective drug delivery vehicle for cancer therapy, supporting the translational potential of these nanostructures [169]. Of particular relevance for HNSCC, cisplatin loading onto DNA origami nanostructures has been successfully achieved, and cytotoxic effects were demonstrated in an HNSCC cell line (FaDu), revealing a sustained and controlled release of cisplatin into target cells [172]. These findings highlight the applicability of DNA origami-based delivery systems in HNSCC-relevant models. Beyond single-drug delivery, DNA origami structures offer additional advantages for improving cancer therapy. DNA origami also enables the simultaneous delivery of multiple therapeutic agents, which may help to overcome the drug resistance mechanisms frequently observed in cancer, including cisplatin resistance in HNSCC [6,168]. The therapeutic effects of DNA origami structures can furthermore be increased through specific engineering which ensures delivery of drugs to specific targets [168]. Importantly, studies using three-dimensional tumor spheroid models have shown that DNA origami nanostructures can achieve deep tumor penetration [173], which is particularly relevant for very solid tumors such as HNSCC.

Beyond their potential as drug delivery systems, DNA origami structures can be exploited to actively alter cellular signaling pathways in cancer (Figure 4C). This was shown, for instance, in one study where DNA origami structures were coupled to a FasL ligand, which led to apoptosis induction in large 3D cancer spheroids [174], highlighting their potential to actively intervene in cellular pathways.

DNA origami allows for the precise intervention of ligand and receptor signaling, which enables the modulation of cellular signaling pathways [175]. Programmable DNA origami nanostructures have been employed to precisely control the nanoscale spatial presentation of integrin-binding peptides and receptor tyrosine kinase (RTK) ligands which enable simultaneous engagement of integrin αvβ6 and receptors such as EGFR, HER2, and c-MET. By systematically varying the ligand spacing, these nanostructures demonstrated that coordinated integrin–RTK crosstalk directly modulates downstream signaling pathways, including focal adhesion kinase, PI3K–AKT, and MAPK/ERK cascades, thereby highlighting how nanoscale ligand organization alone can reshape cellular signaling responses independently of drug delivery [176]. Importantly, this concept expands the functional scope of DNA origami beyond drug carriers toward active regulators of cell signaling, positioning these nanostructures as next-generation tools for precision oncology.

As mentioned before, the tumor microenvironment plays a very important role in HNSCC. DNA origami structures have emerged as highly versatile platforms for modulating immune responses due to their ability to present immune-stimulatory motifs with nanometer precision. Square-block DNA origami scaffolds with optimally spaced CpG oligonucleotides significantly enhance dendritic cell activation, promote Th1 polarization, and amplify both CD8^+^ T-cells and NK cell responses, demonstrating potent immune stimulation and improved therapeutic efficacy [177]. Early studies showed that hollow DNA origami tubes densely decorated with CpG sequences trigger strong innate immune responses through Toll-like receptor 9 engagement, leading to elevated cytokine secretion (e.g., IL-6 and IL-12p70) and immune cell activation without detectable cytotoxicity in primary splenocytes [178]. However, excessive or prolonged immune stimulation may also promote inflammatory toxicity or immune-related adverse events, particularly in anatomically sensitive head and neck tissues. Future nanostructure-based immunomodulatory strategies will therefore need to balance potency with spatial restriction and temporal control. Beyond ligand presentation, DNA origami functionalized with aptamers can be engineered to capture immunosuppressive cytokines such as TGF-β1, facilitating their rapid clearance and thereby enhancing antitumor immune modulation, a strategy that has shown promise in preclinical tumor models [179].

Overall, the high programmability of DNA origami enables multifunctional nanostructures that combine controlled drug delivery with direct modulation of cellular signaling and immune responses, positioning these platforms as powerful tools in cancer biology and therapy. However, their successful clinical translation will critically depend on overcoming current limitations related to structural stability, immunogenicity, and susceptibility to enzymatic degradation under physiological and inflammatory tumor conditions.

### 3.4. Exploiting the Intrinsic Biological Properties of the HNSCC Tumor Microenvironment: Stimuli-Responsive Smart Nanoparticles

**Figure 5:**
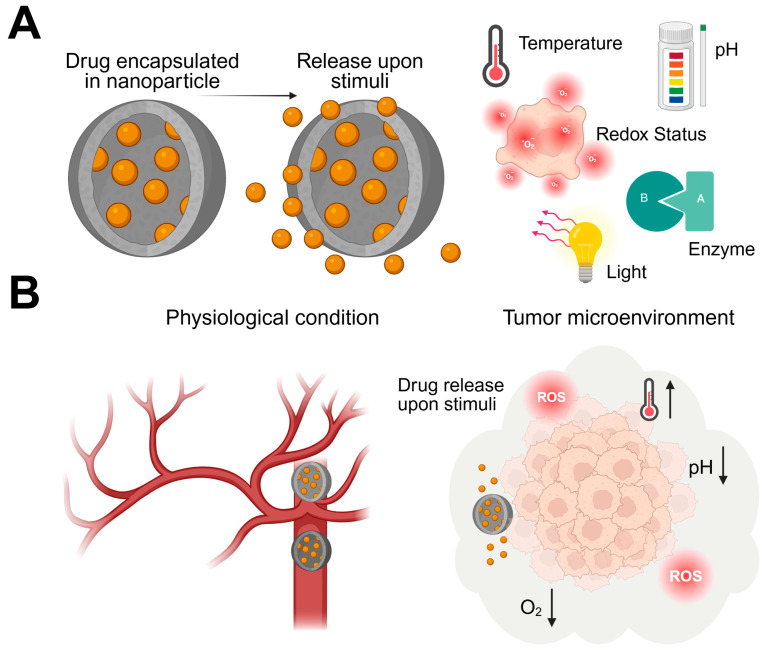
Stimuli-responsive nanoparticle-based drug release. Therapeutic agents are encapsulated within nanoparticles (**A**) and are retained under physiological conditions (**B**). (**A**,**B**) Upon exposure to specific internal or external stimuli, the nanoparticles undergo structural or chemical changes that trigger controlled drug release at the target site. Stimuli include the decreased pH levels in tumor tissues (pH 6.5–7), increases in temperature (ΔT = 1–3 °C), redox status, enzymatic activity, or light. Created in BioRender. Kummer, N. (2026) https://BioRender.com/hdzowjx (accessed on 9 February 2026).

The existing requirements for cancer treatment may be fulfilled through advancements in contemporary drug delivery techniques. In recent times, despite the exploration of numerous drug delivery methods in diverse formats for cancer therapy, polymeric nano-platforms have garnered significant interest owing to their customizable core–shell structures, which can be employed for both physical and chemical applications. Certain innovative designs in nanostructures can prove to be exceptionally beneficial in the fight against various therapeutic and biomedical applications [50,180]. Enhancing drug solubility, ensuring favorable effects on pharmacokinetics, fostering increased permeability, and optimizing retention (EPR) properties are pivotal considerations in the formulation of drug delivery systems. When polymeric nano-platforms are engineered to react to external stimuli, they emerge as exceptionally promising candidates for drug delivery solutions [181]. This unique characteristic of many tumor cells facilitates the preferential accumulation of polymeric nanocarriers of specific dimensions within tumor cells compared to normal tissues [182]. Despite the utilization of polymeric nano-platforms, managing drug release remains a significant challenge. There are particular criteria that these polymeric platforms must meet to enhance efficacy and minimize adverse effects associated with cancer therapies. Polymeric nano-platforms designed to release drug payloads at targeted sites in a controlled manner in response to specific stimuli, such as variations in pH, temperature, and enzymatic activity, are especially attractive (Figure 5A,B). These responsive platforms, known as “smart” polymers, enable drug release to be activated by diverse stimuli. The ability to respond to such triggers enhances the application of nanocarriers and promotes more effective drug delivery to affected areas [182]. Stimuli-responsive nano-platforms exhibit remarkable promise, particularly in the realm of efficient drug delivery systems. By meticulously designing these nano-platforms, one can regulate the release rate of anticancer medications, as well as mitigate adverse side effects and toxicity [183]. In this section, we will examine certain important stimuli-responsive nanostructures.

As previously described, the tumor microenvironment in HNSCC is characterized by many intrinsic features that demarcate the tumor environment from healthy tissues. These features, including the reduced pH and the redox status of the TME, as well as enzyme status, can be exploited for TME-specific delivery of cargo using stimuli-responsive smart nanoparticles (Figure 5, Table 1).

#### 3.4.1. pH-Responsive NPs

Tumor biology exhibits distinctive characteristics, including hypoxemia, reduced carbohydrate levels, low pH, and elevated glutathione content (Figure 1B and Figure 5). Ongoing research into drug delivery systems that react to specific stimuli considers various properties, such as pH [184,185,186,187,188].

These techniques are meticulously crafted to facilitate the release of medication by influencing their conformation. Consequently, alterations in phase occur in response to a specific stimulus agent, while maintaining a minimal drug release ratio throughout the bloodstream. By employing such sophisticated drug delivery methods, the concentration of the drug within the targeted tumor is optimized, achieving success in the therapeutic landscape with minimal side effects. The extracellular pH in tumor cells is significantly lower compared to that of normal tissues (Figure 1B and Figure 5). Healthy tissues maintain a pH of 7.4, whereas tumor cells display an extracellular pH that ranges from 6.5 to 7. This occurrence is linked to the heightened aerobic glycolysis, often known as the Warburg effect. Additionally, when nanostructures penetrate the cells through endocytosis, they encounter different pH environments, particularly pH levels between 5.0 and 6.0 within endosomal structures and between 4.5 and 5.0 in lysosomes [189]. The pH disparities during transitions between these tissues can be harnessed as internal stimuli for drug release [190]. To facilitate a response in the acidic microenvironments of tumor cells, two fundamental recommendations are proposed for the design of polymeric nano-platforms. Firstly, it is essential to employ polymers that possess functional groups acting as proton donors or acceptors in response to changes in environmental pH. Such polymers become deprotonated at physiological pH levels, while they are protonated under acidic conditions, leading to structural damage and alterations in the hydrophobicity of these polymers, which in turn triggers the release of their specific payloads [191,192,193,194].

#### 3.4.2. Temperature-Responsive NPs

Temperature-sensitive materials have primarily been studied for their applications in intelligent drug delivery systems, owing to their phase transition behaviors in response to temperature variations. Temperature can act as both an external and an internal stimulus. When heat originates externally, it is classified as an external stimulus, whereas an increase in temperature that occurs due to the specific location is referred to as an internal stimulus. This distinction is particularly significant, as tumor tissues are known to exhibit slightly elevated temperatures compared to normal tissues, typically ranging from 1 to 3 °C [182,195,196,197] (Figure 1B and Figure 5).

Temperature-sensitive nanostructures have endowed them with a distinctive characteristic during the phase-change event, as the binding and elution processes hinge on minor temperature fluctuations [198]. This exhibited behavior can serve as a mechanism to evaluate the loading and release of various therapeutic agents, functioning as loading and contraction behavior. The drugs are introduced into these thermosensitive nano-platforms at temperatures that fall below the critical solution temperature. Thermosensitive polymers demonstrate a temperature-dependent phase transition, which is characterized by their solubility or insolubility, known as the critical solution temperature (CST). The thermo-transition, which refers to the temperature shift from a more soluble to a less soluble state, is identified as the lower critical solution temperature (LCST). Most temperature-sensitive polymers are engineered to possess this specific property. Below this temperature, the polymer chains expand and dissolve in water. However, in contrast to the polymer chain, the polymer remains undissolved above this temperature. These responsive behaviors to temperature variations render thermosensitive nanomaterials highly appealing for controlled drug delivery applications. Poly(N-isopropylacrylamide) (pNIPA) is a widely utilized thermosensitive polymer due to its low critical solution temperature of approximately 32–34 °C. The slight variations around 32–34 °C lead to rapid and reversible hydration–dehydration phase transitions [182,199,200].

#### 3.4.3. Redox-Responsive NPs

Both tumor cells and their microenvironments exhibit intrinsic differences, resulting in a heterogeneous environment. A prime illustration of this is the disparity in redox potential between normal tissues and tumor cells (Figure 1B and Figure 5). Research suggests that tumor cells maintain elevated levels of intracellular glutathione (GSH) in comparison to their normal counterparts, which exist in a reducing environment. Moreover, it has been noted that oxidative stress manifests in cancer cells, culminating in an increase in ROS (Figure 1B and Figure 5). The variation in redox potential between the oxidizing extracellular space and the reduced intracellular milieu serves as a potential catalyst for the release of therapeutics. In contrast, within reducing environments, structures that contain disulfide bonds undergo reduction to yield thiol groups. Consequently, polymeric nano-platforms attain solubility in the presence of excess glutathione and liberate biologically active factors upon cellular entry [50].

#### 3.4.4. Enzyme-Responsive NPs

Enzymes play a crucial role in numerous biological and metabolic processes, often displaying abnormal expression levels in various disease-related microenvironments, particularly in cancer [201]. In comparison to other stimuli, enzymatic reactions are typically rapid and efficient, occurring under moderate conditions. Furthermore, many enzyme-responsive nanomaterials, which are derived from polymers, liposomes, small organic molecules, and inorganic/organic hybrid materials, can be activated with enhanced specificity, and their biocompatibility is advantageous for clinical applications [201]. To date, several enzyme classes, including proteases and phosphatases, have been identified as biomarkers for both diagnosis and treatment, with many being utilized to create stimuli-responsive nanomaterials for diagnostic purposes, imaging, and drug delivery [202]. Among these enzymes, matrix metalloproteinases (MMPs) are the ones most recognized as stimuli in enzyme-responsive systems, particularly in cancer theranostics. MMPs are zinc-dependent endopeptidases that facilitate the degradation of extracellular matrix (ECM) proteins and the modulation of bioactive molecules on cell surfaces [203]. In cancerous tissues, their expression levels are significantly elevated compared to normal tissues. They can enhance tumor metastasis and invasion due to their capacity to degrade the connective tissue between cells and the lining of blood vessels, thereby allowing tumor cells to escape from their original sites [204]. Based on the differences in expression levels, MMPs have been utilized as triggers, leading to the development of various nanomaterials for diverse applications [205,206,207].

#### 3.4.5. Dual- and Multi-Stimuli-Responsive NPs

In recent years, polymeric nanoparticles have emerged as an incredibly promising and effective technological platform for the precise and controlled delivery of drugs. When considered as carrier systems, optimal nanoparticles are expected to ensure that the drug reaches the designated pathological site and/or target cells without any leakage during transport, while also enabling rapid drug release at the site of action and possessing high drug-loading capacities. To achieve this, a range of “smart” polymeric nanoparticles that release drugs in response to internal or external triggers, such as pH level, redox states, temperature parameters, magnetic fields, and light, have been actively studied and investigated. These stimulus-sensitive nanoparticles have been reported in the literature to exhibit varying degrees of improvement in in vitro and/or in vivo drug release profiles. To further enhance drug delivery efficiency, innovative dual- and multi-stimulus-responsive polymeric nanoparticles have been formulated to respond to combinations of two or more signals, including pH/temperature, pH/redox, pH/magnetic field, temperature/reduction, dual pH, pH and diols, temperature/magnetic field, temperature/enzyme, temperature/pH/redox, temperature/pH/magnetic, pH/redox/magnetic, temperature/redox/guest molecules, and temperature/pH/guest molecules. Specifically, these combined and multi-response systems occur either simultaneously in the pathological site or sequentially, from nanoparticle preparation and transport pathways to cellular compartments. Dual- and multi-stimulus-responsive polymeric nanoparticle systems have demonstrated unparalleled control over drug delivery and release, enhancing anticancer efficacy in vitro and/or in vivo. With their programmed capabilities for target-specific drug delivery, dual- and multi-stimulus-responsive nanoparticle drug formulations hold enormous potential for targeted cancer therapy [180,208,209,210,211,212]. Although multi-stimuli-responsive nanoparticles represent an attractive concept for precision therapy, achieving reliable spatial and temporal control of multiple triggers remains challenging in the highly heterogeneous and dynamically evolving HNSCC TME. Variations in pH, redox status, oxygenation, and enzymatic activity within and between tumors may limit synchronized activation. Future advances will therefore require robust, hierarchically programmed systems and improved patient-specific stratification to ensure predictable and clinically relevant responses (Table 1).

## 4. Conclusions and Future Directions

Ancient nanostructures are of more than historical curiosity. Their natural compatibility with biological systems, ability to carry diverse therapeutic agents, and relative safety profiles position them as a useful starting point for modern cancer nanomedicine. Yet even the most promising platforms face the same bottlenecks that have limited progress in head and neck cancer for decades: inconsistent delivery to the tumor core, limited access to intracellular targets, and a lack of control over when and where therapeutic cargo is released. These problems will not be solved by inventing more particle types in isolation. What is needed is a shift toward integrated systems that can recognize tumor cells, move through complex tissue, and adjust their activity within the local biochemical environment.

Several priorities emerge from this perspective. First, drug delivery systems must be coupled to mechanisms that sense and respond to the dynamic conditions of the tumor microenvironment. Ancient nanostructures already offer useful scaffolds for this, but their behavior in inflamed, fibrotic or hypoxic regions of HNSCC remains poorly understood. A stronger connection between nanomaterial design and real-time imaging or mechanobiological profiling would allow materials to be adapted iteratively rather than fixed at the time of formulation. Second, intracellular targeting needs to become routine rather than exceptional. Many of the most important drivers of therapy resistance in HNSCC operate inside the cell and are not easily addressed by surface-binding approaches. Strategies that combine molecular recognition units such as nanobodies with biogenic carriers or DNA-based structures capable of entering the cytoplasm or nucleus could open therapeutic space that is currently inaccessible.

It is also important not to assume that natural materials are automatically safe. Plant-derived vesicles and biopolymer systems such as alginate or collagen vary widely between sources, preparation methods and batches. Their pharmacology and immunology need to be characterized with the same rigor applied to synthetic systems. Without this, the advantages of biocompatibility may remain largely anecdotal and limit regulatory acceptance. The field will benefit from systematic toxicology programs that address batch variation, stability, and interaction with standard-of-care drugs such as cisplatin or radiotherapy.

A second challenge concerns manufacturing and scalability. Even the most elegant systems will remain experimental curiosities unless they can be produced consistently and at relevant volume. Ancient nanostructures may have an advantage here, since many of their components already have existing supply chains and established safety records, but their nanoscale formulations rarely meet regulatory expectations for stability, purity and traceability. Future success will require closer interaction between materials scientists, formulation chemists and clinical pharmacologists to establish manufacturing routes that preserve biological activity without compromising reproducibility. Importantly, despite the rapid expansion of preclinical research on nanostructured systems in HNSCC, robust clinical evidence remains scarce. To date, most engineered exosomes, DNA-based nanostructures, and stimulus-responsive platforms have not progressed beyond early-stage experimental or proof-of-concept studies. Large, well-controlled clinical trials evaluating their therapeutic efficacy and long-term safety are largely lacking. Major obstacles to clinical translation include limited reproducibility, insufficient understanding of in vivo pharmacokinetics, challenges in large-scale manufacturing, and uncertainties regarding long-term immunogenicity and off-target effects. In addition, the heterogeneity of HNSCC and its treatment regimens complicates patient stratification and trial design. Addressing these translational barriers will be essential for moving nanostructured therapeutics from experimental models into routine clinical practice.

Although critical challenges such as incomplete spatial control, redox heterogeneity, and the risk of immune-related toxicity are not yet fully resolved, nanostructure-based approaches already offer a more refined level of biological modulation than conventional chemotherapeutics, which largely rely on systemic exposure and unspecific cytotoxicity.

Looking ahead, the approaches with the highest potential are likely to be hybrid systems that combine complementary functions: recognition and targeting through nanobodies or aptamer-like modules, structural stability and tolerability through biogenic materials, and conditional release through pH, redox or enzymatic cues. DNA origami adds an additional level of spatial precision and may allow defined loading of multiple agents, which is directly relevant to overcoming drug resistance in HNSCC. The most promising innovations therefore lie not in single materials but in their coordination. If this coordination can be achieved, nanomedicine may finally move beyond incremental gains in drug delivery toward therapies that engage with the complexity of head and neck cancer with equal precision. A final consideration is strategic prioritization. The field has produced an abundance of material classes and proof-of-concept studies, yet relatively few efforts have advanced far enough to reveal why therapies ultimately fail or succeed in patients. Progress will depend on selecting problems that are both scientifically tractable and clinically urgent—such as improving drug penetration into hypoxic tumor nests, suppressing adaptive resistance after radiotherapy, and achieving durable intracellular Survivin or STAT3 depletion. Ancient nanostructures, with their intrinsic tolerance and tunable properties, could be used as stable chassis on which these targeted mechanisms can be mounted, while DNA origami and nanobody-based systems contribute the precision required to strike specific vulnerabilities. If future studies align material design with defined bottlenecks in HNSCC biology rather than broad ideals of “targeted delivery,” the field may converge on modular platforms that can be adapted across tumor subtypes. This focus, rather than diversification alone, is likely to determine which nanotechnologies eventually make a clinical impact.

## Figures and Tables

**Table 1:** Comparative overview of major nanostructure platforms for targeted drug delivery and therapeutic modulation in head and neck squamous cell carcinoma (HNSCC).

Nanostructure Platform	Targeting Capability	Drug-/Cargo-Loading Capacity	Controllable Release	Biocompatibility & Immunogenicity	Clinical Translation Potential
Nanobodies	Very high (epitope-specific; surface & intracellular targets)	Low–moderate (primarily conjugates or fusion-based)	Limited (depends on conjugation strategy)	High biocompatibility; low immunogenicity	Moderate–high (several imaging and therapeutic formats in clinical evaluation)
Engineered exosomes	High (surface ligand engineering; intrinsic cell–cell communication)	High (proteins, RNA, small molecules, chemotherapeutics)	Moderate (cargo-dependent; limited stimulus-responsiveness)	Excellent biocompatibility; minimal immunogenicity	Moderate (rapidly advancing but still largely preclinical)
DNA origami nanostructures	High (precise ligand spatial organization; receptor patterning)	High (drugs, ligands, immune motifs; multi-cargo)	High (programmable release; spatial control)	Generally good; immune activation depends on design	Low–moderate (strong preclinical promise, early translational stage)
Stimulus-responsive nanoparticles	Moderate–high (ligand-functionalized)	High	High (environment-triggered release)	Variable; may induce immune responses	Moderate–high (several systems in clinical trials)

## Data Availability

No new data were created or analyzed in this study.
